# The X-linked 1.688 Satellite in *Drosophila melanogaster* Promotes Specific Targeting by Painting of Fourth

**DOI:** 10.1534/genetics.117.300581

**Published:** 2017-12-12

**Authors:** Maria Kim, Samaneh Ekhteraei-Tousi, Jacob Lewerentz, Jan Larsson

**Affiliations:** Department of Molecular Biology, Umeå University, SE-90187, Sweden

**Keywords:** Painting of fourth, dosage compensation, heterochromatin, epigenetics, *Drosophila melanogaster*

## Abstract

Repetitive DNA, represented by transposons and satellite DNA, constitutes a large portion of eukaryotic genomes, being the major component of constitutive heterochromatin. There is a growing body of evidence that it regulates several nuclear functions including chromatin state and the proper functioning of centromeres and telomeres. The 1.688 satellite is one of the most abundant repetitive sequences in *Drosophila melanogaster*, with the longest array being located in the pericentromeric region of the X-chromosome. Short arrays of 1.688 repeats are widespread within the euchromatic part of the X-chromosome, and these arrays were recently suggested to assist in recognition of the X-chromosome by the dosage compensation male-specific lethal complex. We discovered that a short array of 1.688 satellite repeats is essential for recruitment of the protein POF to a previously described site on the X-chromosome (*PoX2*) and to various transgenic constructs. On an isolated target, *i.e.*, an autosomic transgene consisting of a gene upstream of 1.688 satellite repeats, POF is recruited to the transgene in both males and females. The sequence of the satellite, as well as its length and position within the recruitment element, are the major determinants of targeting. Moreover, the 1.688 array promotes POF targeting to the *roX1*-proximal *PoX1* site in *trans*. Finally, binding of POF to the 1.688-related satellite-enriched sequences is conserved in evolution. We hypothesize that the 1.688 satellite functioned in an ancient dosage compensation system involving POF targeting to the X-chromosome.

THE specific targeting and differential loading of transcription factors and epigenetic regulatory complexes are vital for proper gene regulation, but the rules that dictate specific targeting are poorly understood. Sex chromosome dosage compensation is widely used as a model system for this phenomenon because it requires chromosome-specific targeting and regulation. In *Drosophila melanogaster*, a twofold increase in gene expression restricted to the single male X-chromosome is achieved by a combination of a general buffering effect exerted on monosomic regions or chromosomes (Stenberg *et al.* 2009; Zhang *et al.* 2010; Lundberg *et al.* 2012), and an increase in expression from the male X-chromosome mediated by the male-specific lethal (MSL) complex (Hamada *et al.* 2005; Deng *et al.* 2009; Prestel *et al.* 2010; Stenberg and Larsson 2011).

An important unanswered question relates to how the MSL complex correctly targets the ∼1000 active genes on the male X-chromosome. Targeting is thought to be initiated by sequence-influenced binding of the complex to 200–300 nucleation sites on the X-chromosome. These sites are low-complexity GA-rich motifs termed chromatin entry sites, high-affinity sites, or PionX (Alekseyenko *et al.* 2008; Straub *et al.* 2008, 2013; Villa *et al.* 2016). The nucleation is stabilized by the zinc finger protein CLAMP (Soruco and Larschan 2014; Kuzu *et al.* 2016), and extends to neighboring genes via a process dependent on active transcription, the concentration of the MSL complex, affinity levels, small interfering RNAs, and sequence composition (Stenberg and Larsson 2011; Philip *et al.* 2012; Straub *et al.* 2013; Menon *et al.* 2014). Notably, the nucleation sites are modestly enriched on the X-chromosome and have limited predictive power. It is generally accepted that male flies exhibit increased expression of X-linked genes, but the molecular mechanisms responsible for this are unknown. Suggested mechanisms, which are the subject of ongoing debate (Ferrari *et al.* 2013, 2014; Straub and Becker 2013), include increased transcriptional initiation (Conrad *et al.* 2012; Vaquerizas *et al.* 2013), increased elongation (Larschan *et al.* 2011; Prabhakaran and Kelley 2012), and an inverse dosage effect (Sun *et al.* 2013).

An additional chromosome-specific compensatory mechanism active in *Drosophila* involves the protein Painting of fourth (POF), which specifically targets the fourth chromosome (Larsson *et al.* 2001; Johansson *et al.* 2007a, 2012). POF binds transcribed genes with a 3′ bias; however, this preference is mainly explained by a strong bias toward exon sequences and the fact that exon densities tend to be higher at the 3′ ends of genes (Johansson *et al.* 2007b). MSL binding has also been described as showing a 3′ bias in binding along transcribed genes (Alekseyenko *et al.* 2006; Gilfillan *et al.* 2006; Gelbart *et al.* 2009). Similarly, this bias was more recently argued to be caused by an exon bias (Straub *et al.* 2013). POF binds to nascent RNA from actively transcribed genes on the fourth chromosome and increases their expression (Johansson *et al.* 2012). The magnitude of this increase is comparable to that induced by the MSL complex on the male X-chromosome, and is sufficient to allow the survival of haplo-4 flies (Johansson *et al.* 2007a, 2012; Deng and Meller 2009; Stenberg *et al.* 2009; Zhang *et al.* 2010). The fourth chromosome has several unique characteristics: it is the smallest chromosome in the *Drosophila* genome, enriched in repetitive DNA, and can (in principle) be considered heterochromatic across its entire length; more specifically, it forms HP1-enriched chromatin as previously defined (Filion *et al.* 2010; Kharchenko *et al.* 2011). This means that the fourth chromosome is enriched not only in the heterochromatin protein HP1, but also in specific histone modification markers of heterochromatin, *e.g.*, methylated H3K9 (Riddle *et al.* 2011; Figueiredo *et al.* 2012). Consequently, one might expect genes on the fourth chromosome to be repressed. However, they are actually expressed at similar or higher levels than genes on other chromosomes, indicating that they have adapted to function in their repressive milieu (Haddrill *et al.* 2008; Johansson *et al.* 2012).

The fourth chromosome is related to the X-chromosome, and evolutionary studies have shown that sex chromosomes do not inevitably represent terminal stages in chromosomal evolution; in fact, the fourth chromosome was ancestrally an X-chromosome that reverted to an autosome (Vicoso and Bachtrog 2013, 2015). This supports our hypothesis that POF originated as an ancestral dosage compensation system with a stimulatory function, and became trapped on the fourth chromosome when the latter reverted to being an autosome. POF has been considered to require an intact fourth chromosome for targeting; even translocations of entire banded segments of the fourth chromosome are not targeted unless they are placed under special conditions. The chromosome 4-specific recognition by POF could thus not be explained by an analogous mechanism involving high-affinity sites and spreading (Johansson *et al.* 2007a). However, we recently showed that POF specifically targets two loci on the X-chromosome, *PoX1* and *PoX2* (POF-on-X). *PoX1* and *PoX2* are located close to the *roX1* and *roX2* genes, and therefore provide an additional evolutionary link between the two targeting systems in *D. melanogaster* (Lundberg *et al.* 2013). To better characterize the requirements for chromosome-specific identification and targeting, and to determine whether POF originated as an ancient dosage compensation factor, we decided to investigate the targeting of POF to *PoX2* by constructing and analyzing transgenic POF-recruitment modules. We present a stand-alone POF-recruiting module and show that POF targeting to *PoX2* depends on repeated 1.688 satellite sequence blocks located downstream of expressed genes. Importantly, the 1.688 satellite is enriched ∼50-fold on the X-chromosome compared to autosomes, and has been suggested to be an X-chromosome identifier (Waring and Pollack 1987; Kuhn *et al.* 2012; Gallach 2014, 2015). We hypothesize that POF, together with the 1.688 satellite, functioned in an ancient dosage compensation system involving direct targeting to the X-chromosome.

## Materials and Methods

### Fly strains, transgenes, and genetic crosses

Flies were cultivated and crossed at 25° in vials containing potato mash-yeast-agar. The duplication of the *PoX2* locus used was *Dp*(*1*;*3*)*DC246*, *PBac*[*y^+mDint2^ w^+mC^ DC246*]*VK00033* (102 kb inserted at 3L:65B2 covering the two genes *SelG* and *CG1840* in *PoX2*) (Venken *et al.* 2010). To generate transgenes, genomic fragments were amplified using Long PCR Enzyme Mix (Thermo Fisher Scientific) with *Oregon R* genomic DNA as the template. The transgenic constructs were cloned into the *P*[*w^+^ attB*] vector and the same *attP* docking site (3L:65B2) was used for all transgenes. Embryo microinjection into the *Bl9750* strain was performed by BestGene. *Oregon R* was used as the wild-type strain. More detailed cloning procedures and the primer sequences used in this work are presented in the Supplemental Material, Table S1 in File S1.

### DNA-FISH and immunostaining of polytene chromosomes

Immunostaining of polytene chromosomes was essentially as described previously (Johansson *et al.* 2012). We used primary antibodies against POF raised in rabbit, diluted 1:500 (Larsson *et al.* 2004), or raised in chicken, diluted 1:100; HP1a (rabbit PRB291C, Covance) diluted 1:400; and rabbit anti-MSL1 (1:400) from Mitzi Kuroda (Harvard Medical School). Goat or donkey anti-rabbit, anti-chicken, and anti-mouse antibodies conjugated with Alexa-Fluor555 or AlexaFluor488 (1:500; Molecular Probes, Eugene, OR) were used as secondary antibodies. DNA-FISH combined with immunostaining on polytene chromosomes was performed according to a standard protocol (Lavrov *et al.* 2004). The probe against the *sicily* gene was PCR-amplified using *D. yakuba* genomic DNA as a template, cloned into the *pCR2.1-TOPO* vector (Thermo Fisher Scientific), excised with *Bam*HI, and labeled with the BioNick DNA Labeling System (Life Technologies). Antibodies for the detection of DNA probes were mouse anti-biotin (1:500; Jackson ImmunoResearch) and goat anti-mouse labeled with AlexaFluor488 as secondary antibody. Preparations were analyzed using a Axiophot microscope equipped with a KAPPA DX20C CCD camera (Zeiss [Carl Zeiss], Thornwood, NY). For comparisons of targeting between different genotypes, the protocol was run in parallel and nuclei with clear cytology were chosen on the basis of DAPI staining and photographed. At least 20 nuclei per slide were used in these comparisons and at least five slides per genotype. Each genotype was classified into one of three groups based on the degree of targeting observed on the corresponding slides. For group (+), targeting was seen on all slides and ≥ 20% of nuclei exhibited targeting. For group (±), targeting was seen on < 20% of the slides, and in these slides < 20% of nuclei were targeted. For group (−), no targeting was observed and ≥ 10 good slides were analyzed.

### Proximity ligation assay (PLA) on polytene chromosomes

To probe potential interactions between POF and MSL3 in *D. ananassae*, we used PLA essentially as described previously (Lindehell *et al.* 2015). The primary antibodies were goat anti-MSL3 and rabbit anti-POF; the corresponding PLA probes were rabbit plus and goat minus (Olink Biosciences). For the negative controls, we used only one of the primary antibodies and the PLA probes as above.

### Generating the PoX2^Df1.688^ deletion strain

A stable line with clustered regularly interspaced short palindromic repeat (CRISPR)/Cas9 deletion of the *1.688^PoX2^* repeat was generated with the approach suggested by Kondo and Ueda (2013). Briefly, a fly strain expressing two guiding RNAs from one transgene was established and crossed with a strain expressing Cas9. The male progeny were crossed individually in two subsequent generations, and the mutant strain was selected by PCR followed by sequencing. Sequences of the guiding RNA oligos are provided in Table S2 in File S1.

### Detection of transcription readthrough by RT-PCR

Total RNA was isolated from five pairs of salivary glands of third instar females, reverse-transcribed with iScript Reverse Transcription Supermix (Bio-Rad, Hercules, CA), treated with RNAse-free DNase I (New England Biolabs, Beverly, MA), and analyzed by PCR with Phire Hot Start II DNA Polymerase (Thermo Fisher scientific). The PCR primers used are listed in Table S3 in File S1.

### Satellite distribution

Basic local alignment search tool (BLAST) searches with the *1.688^PoX2^* sequence, the *1.688^Xhet^* sequence, and the *D. ananassae* satellite 191 (Gallach 2014) were used to determine the cytological location of satellite sequences. Default parameters were used except for the word_size parameter, which was set to 5. To qualify a valid alignment, BLAST alignments were filtered by those alignments that covered ≥ 33% of the query sequence length. We defined 1.688 blocks as a chain of alignments that appeared within 1000 bp of each other. To calculate randomized distributions, the blocks’ locations were randomized back onto the X-chromosome using 1000 iterations with and without allowing blocks into exon sequences.

### Data availability

The authors state that all data necessary for confirming the conclusions presented in the article are represented fully within the article.

## Results

### Identifying a POF recruitment element

**Figure 1:**
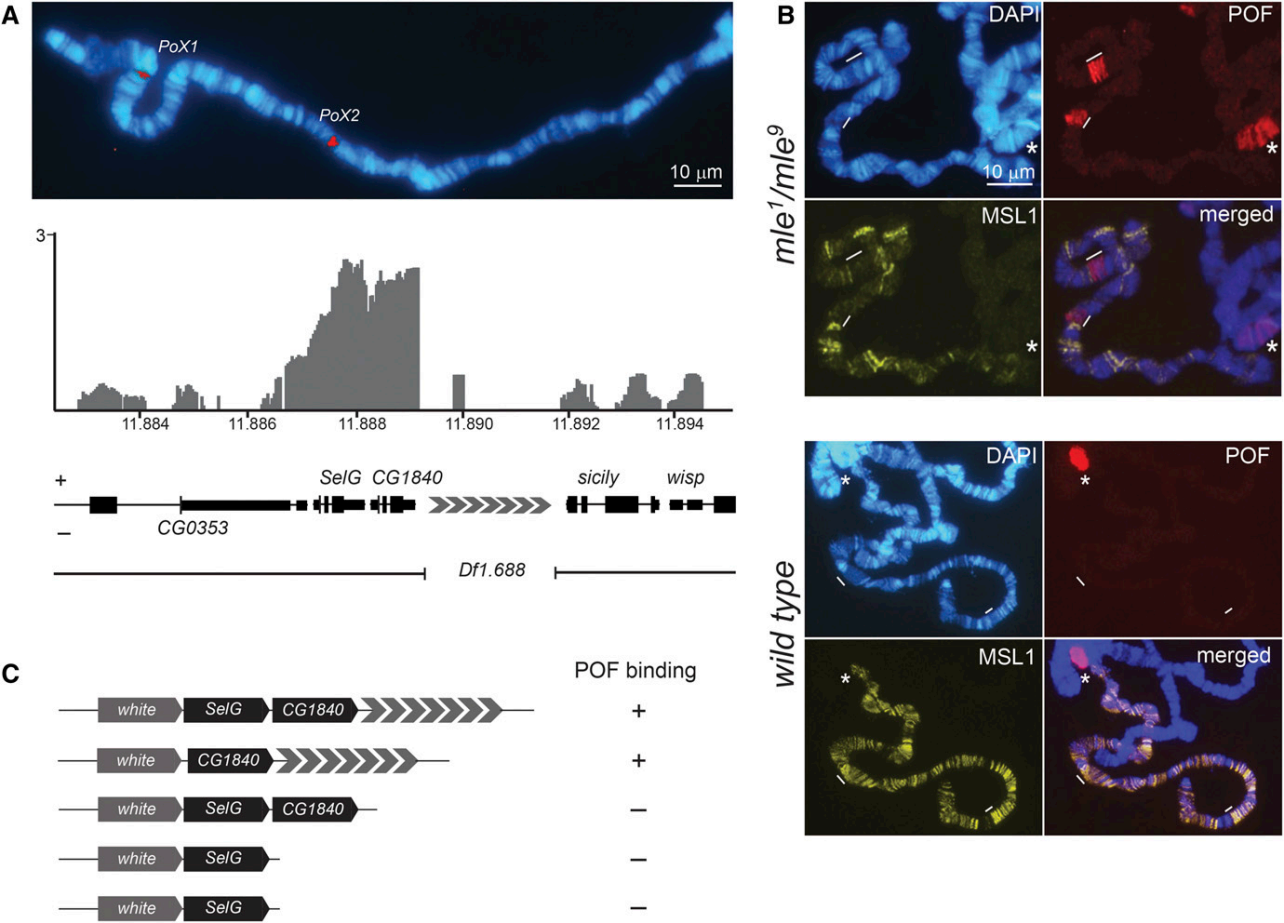
*PoX2* harbors a POF recruitment module. (A) Immunostaining of POF on the X-chromosome in wild-type females. The chromatin immunoprecipitation (ChIP) profile of POF in the wild-type is shown below (Lundberg *et al.* 2013). The plots show the mean enrichment values expressed as log2 ChIP ratios. Numbers on the *x*-axis denote chromosomal position in megabases, and the *y*-axis shows ChIP enrichments. The lack of enrichment downstream of *CG1840* is caused by this region being repeat masked in the analysis. The genes are indicated; genes expressed from left to right are labeled (+) and those expressed in the opposite direction are labeled (−). The extent of the *PoX2^Df1.688^* deletion is indicated. (B) POF is targeted to *PoX1* and *PoX2* in males in a *mle* mutant background, but not in wild-type. *PoX* sites are indicated by strokes and the fourth chromosome by a star. Note the strong and widespread POF sites on the X-chromosome in *mle* mutants, not overlapping with the MSL1 staining. (C) Schematic illustration of transgenic constructs with their targeting properties indicated. The same *attP* docking site (3L:65B2) was used for all transgenes. +, targeting was seen on all slides (*n* ≥ 5) and ≥ 20% of nuclei exhibited targeting; −, no targeting was observed and ≥ 10 good slides were analyzed.

POF binds with high specificity to the fourth chromosome in both males and females (Larsson *et al.* 2001; Johansson *et al.* 2007a,b; Figueiredo *et al.* 2012), and two loci on the X-chromosome, *PoX1* and *PoX2* (POF-on-X), are targeted, but only in females (Lundberg *et al.* 2013) (Figure 1A). Since *PoX2* is a robust but isolated POF target, we set out to determine what is required for its targeting. We have previously made a transgenic construct consisting of the gene *CG1840* and its 2600-bp downstream region, which functioned as a stand-alone targeting module. Notably, in contrast to the endogenous loci, the *P*[*w^+^ CG1840_7×1.688*] transgene is also targeted in males. A 102-kb duplication of the *PoX2* site on the 3L chromosome has been shown to bind POF or the MSL complex in females and males, respectively (Lundberg *et al.* 2013). Furthermore, robust targeting to the endogenous *PoX1* and *PoX2* loci is observed in *mle* mutant but not wild-type males, strongly suggesting that the lack of targeting to the endogenous *PoX* loci in males is caused by the presence of the MSL complex (Figure 1B). Additional transgenes revealed that the region downstream of *CG1840* is essential for targeting in both sexes (Figure 1C). We analyzed the sequence and found seven repeats of the 1.688 satellite. The repeat is 360 bp long and shows ∼70% identity to the 1.688 consensus sequence from the pericentromeric 1.688 block. The *1.688^PoX2^* sequence is more similar to 1.688 repeats on the X-chromosome and nearby repeats (in cytological terms) than to those that are located on other chromosomes or more cytologically distant (Figure S1 in File S1). Importantly, the targeting to transgenes appears to be binary: one or both *PoX* sites exhibit distinct targeting, with POF either being present or absent and no apparent intermediate states being identifiable. In addition, HP1a is only targeted to the transgene when POF is targeted, *i.e.*, HP1a is not targeted to the *1.688^PoX2^* repeat in the absence of POF. We conclude that *CG1840_7×1.688* is a novel POF recruitment module. In the context of a male X-chromosome, MSL outcompetes POF. However, as an isolated target, POF is recruited to the transgene but not MSL, suggesting that POF is the primary targeting protein of genes in the vicinity of the 1.688 satellite.

### The 1.688 satellite is essential for targeting

**Figure 2:**
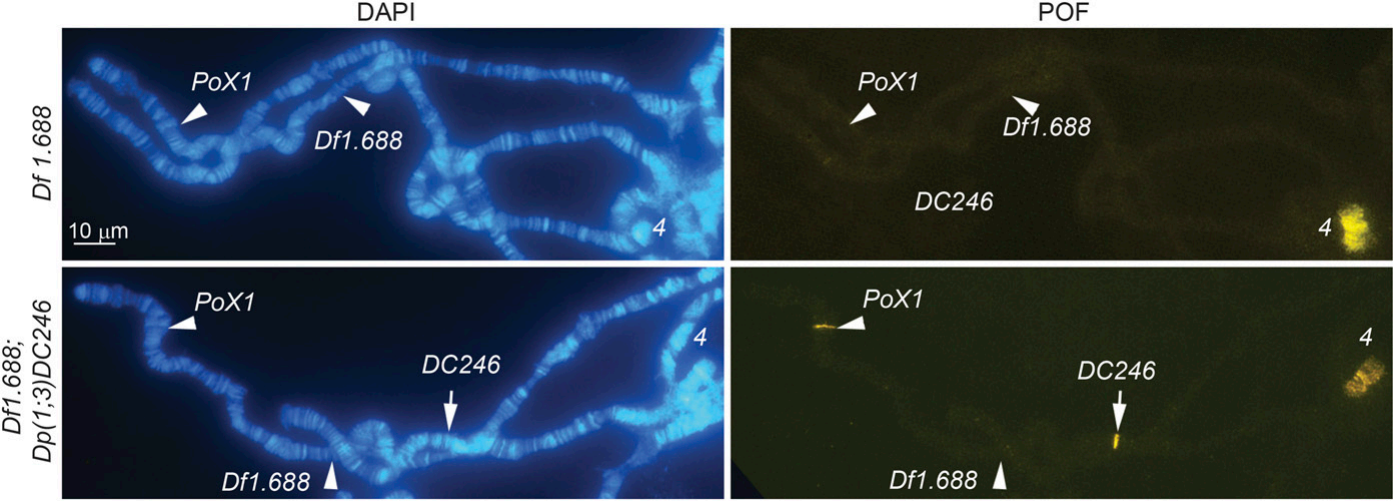
*PoX2* supports targeting to *PoX1* in *trans*. In a *PoX2^Df1.688^* female, POF targeting to *PoX1* is lost in almost all nuclei. Targeting to the endogenous *PoX1* (but not endogenous *PoX2*) is rescued by a duplication of the *PoX2* region on the 3L chromosome, *Pox2^Df1.688^*; *Dp*(*1*;*3*)*DC246*.

Our results show that an array of 1.688 satellite sequences downstream of *CG1840* is sufficient for robust targeting. We next asked whether the endogenous *1.688^PoX2^* array is essential for POF targeting to this X-chromosomal locus. To answer this question, we used the CRISPR/Cas9 technique to generate a deletion mutant. A perfect deletion of *1.688^PoX2^* was generated, *Pox2^Df1.688^* (Figure 1A). As expected, we detected no targeting of POF to *PoX2* in this mutant line. Intriguingly, although *PoX1* targeting was not completely lost, it was extremely rare, being observed in only 1–2 nuclei on two slides out of 20 that were examined (Figure 2). POF targeting to the endogenous *PoX1* (but not the endogenous *PoX2*) locus is rescued by a transgenic duplication of the *PoX2* locus (102 kb) inserted in the *attP* docking site in genomic region 3L:65B2. We conclude that the *1.688^PoX2^* satellite is essential for targeting of POF to *PoX2* and also stabilizes weak X-chromosome targets in *trans*, *e.g.*, *PoX1*.

The 1.688 satellite is the most abundant satellite DNA in the *D. melanogaster* genome; it is estimated to account for ∼5% of total embryonic DNA (Ashburner *et al.* 2005; Krassovsky and Henikoff 2014) and believed to form a multi-megabase pair block of heterochromatin on the proximal X-chromosome also known as *Zhr* (Ferree and Barbash 2009). Therefore, we asked whether the presence of the pericentromeric 1.688 satellite block affected targeting. We compared the targeting to the *PoX2* locus in the wild-type and *Zhr^1^* mutants lacking the pericentromeric 1.688 satellite block. No influence from the pericentromeric block on targeting was observed. We conclude that the *CG1840_7×1.688* sequence in the *PoX2* locus functions as a stand-alone targeting element independent of the megabase pair block of 1.688 satellite on the proximal X-chromosome known as *Zhr*.

### A functional recruitment module comprising a gene and 1.688

**Figure 3:**
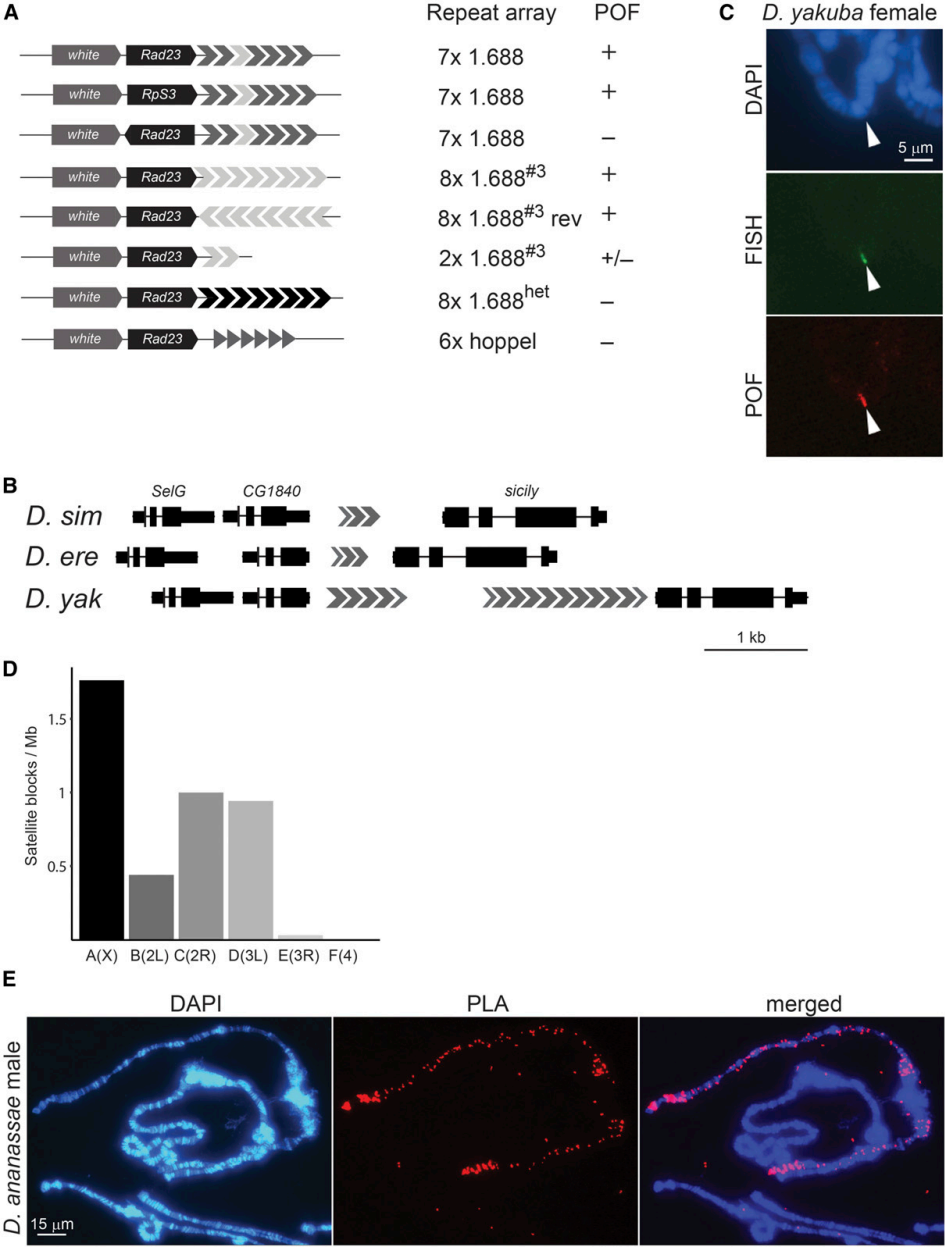
POF binding to transgenic POF recruitment modules. (A) Schematic illustrations of POF recruitment constructs and their targeting properties when inserted at position 3L:65B. (+) targeting was seen on all slides (*n* ≥ 5) and ≥ 20% of nuclei exhibited targeting. (±) targeting was seen on < 20% of the slides (*n* ≥ 20) and in these slides, < 20% of nuclei were targeted. (−) no targeting was observed and ≥ 10 good slides were analyzed. The targeting properties were identical in males and females. (B) Genomic organization of the *PoX2* locus in *D. simulans*, *D. erecta*, and *D. yakuba*. The 1.688-related repeats are indicated by gray arrowheads. (C) FISH immunostaining of *sicily* (green) and POF (red) in *D. yakuba* females. (D) Frequency distribution of the *D. ananassae* 191 repeat on Mullers elements A–F with the corresponding chromosome annotations in *D. melanogaster* within brackets. (E) *In situ* proximity ligation assay (PLA) verifies the close proximity of POF and the MSL complex in *D. ananassae* males, as illustrated by signals on the left and right arms of the X-chromosome obtained using probes for POF and MSL3.

To further define the requirements for a functional targeting element, we generated an array of transgenes, all inserted in the same *attP* docking site (3L:65B2), and analyzed the targeting of POF on salivary gland polytene chromosomes. Four different genes with their endogenous promoter regions were inserted upstream of the repeat block: *CG1840* alone and in tandem with its paralogue *SelG* from the X-chromosome, *Rad23* from chromosome 4, and *RpS3* from chromosome 3. We observed robust targeting in all cases, suggesting that the targeted genes do not harbor specific sequence information. We next asked whether the number of repeats, orientation, or minor differences between the repeats influence targeting. A transgene where the endogenous 1.688 array was replaced with eight identical copies of the 360 bp third repeat (*8 × 1.688^#3^*) was robustly targeted. In fact, even a transgene with only two repeats (*2 × 1.688^#3^*) exhibited targeting, albeit at a lower frequency (Figure 3A). Inverting the targeted gene disrupted targeting, but inverting the satellite block did not affect its targeting capacity. To determine whether repeats in general support targeting, we constructed transgenes with eight copies of the 1.688 satellite sequence from the pericentromeric region (*8 × 1.688^Xhet^*) and a transgene with six copies of the *Hoppel* element (*6 × hoppel*), which is highly enriched on the fourth chromosome (Coelho *et al.* 1998). No targeting was detected with these constructs, indicating that there is sequence information in the *PoX2* euchromatic *1.688^PoX2^* satellite repeat that is absent in the *1.688^Xhet^* pericentromeric repeat, and that the euchromatic 1.688 repeat is not direction-dependent.

We next asked whether the genomic organization of the *PoX2* locus is conserved in related species. In the three related species *D. simulans*, *D. erecta*, and *D. yakuba*, the genes *CG1840* and *sicily* are separated by arrays of 1.688-related sequences (Figure 3B). In *D. yakuba*, this region functions as a robust target of POF (Figure 3C), suggesting functional conservation. In some species, such as *D. busckii* and *D. ananassae*, POF targets the male X-chromosome; in those species, POF colocalizes with H4K16ac and the MSL complex, respectively (Larsson *et al.* 2004; Stenberg and Larsson 2011). Therefore, we hypothesized that POF originated as a dosage compensation system. In keeping with a role in defining chromosome identity, it has been shown that the X-chromosome and the F-element (fourth chromosome) are overpopulated by certain repetitive elements in several *Drosophila* species (Gallach 2014) and that the X-chromosome in *D. ananassae* is highly enriched in a 191-bp satellite (Figure 3D) (Gallach 2014). Since POF in *D. ananassae* colocalizes with the MSL complex, we used a proximity ligation assay method to determine whether POF and MSL are in close proximity, *i.e.*, whether POF in this species is part of the MSL complex. POF and MSL colocalize in *D. ananassae*, and the strong PLA signal on the left and right arms of the *D. ananassae* X-chromosome (Figure 3E), but not in the negative control (Figure S2 in File S1), suggesting that POF in this species is included in the MSL complex. These results imply that POF may have an ancient role in dosage compensation and even forms part of the MSL complex in some species.

### Transcription of 1.688 repeats

**Figure 4:**
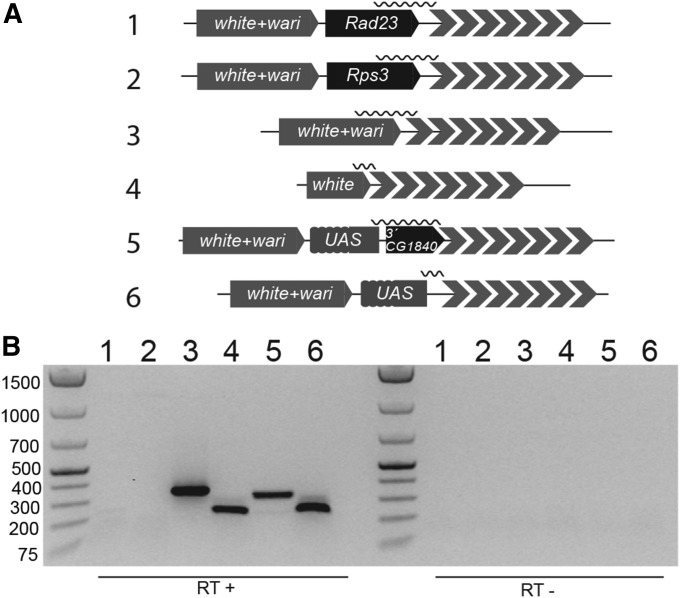
Nontargeted transgenes show high transcription readthrough. (A) Schematic representations of the transgenic constructs shown in (B). Wavy lines indicate the RT-PCR product. For transgenes 5 and 6, transcription was induced with an actin5C-Gal4 driver. (B) Agarose gel showing the use of RT-PCR to detect transcription readthrough. Numbers above the gel correspond to the transgenes shown in (A). Control reactions without reverse transcriptase are shown to the right.

Our data suggest that a POF-binding module consists of a gene and a 1.688 satellite array downstream. However, a transgene construct featuring *mini-white* as the upstream gene is an exception since it is not targeted by POF. Therefore, we hypothesized that transcription readthrough may be involved in the targeting mechanism, and that the strength of this transcription depends on the gene’s properties. To exclude the possible influence of the *wari* insulator located immediately downstream of *mini-white* (Chetverina *et al.* 2008), we generated an additional transgene without *wari* (Figure 4A). Strikingly, these two transgenes differ from our targeted transgenes in that strong and reproducible transcription readthrough was detected (Figure 4B, lanes 3 and 4). When even stronger transcription was induced by a upstream activating sequence promoter placed 9- or 282-bp upstream of the *CG1840* transcription end (Figure 4B, lanes 5,6), we also detected no POF targeting. It remains to be determined whether the transcription termination in these cases is required or is a consequence of targeting. Regardless, strong transcription readthrough into the satellite correlates with an absence of POF targeting.

### Genomic distribution of 1.688 repeats:

**Figure 5:**
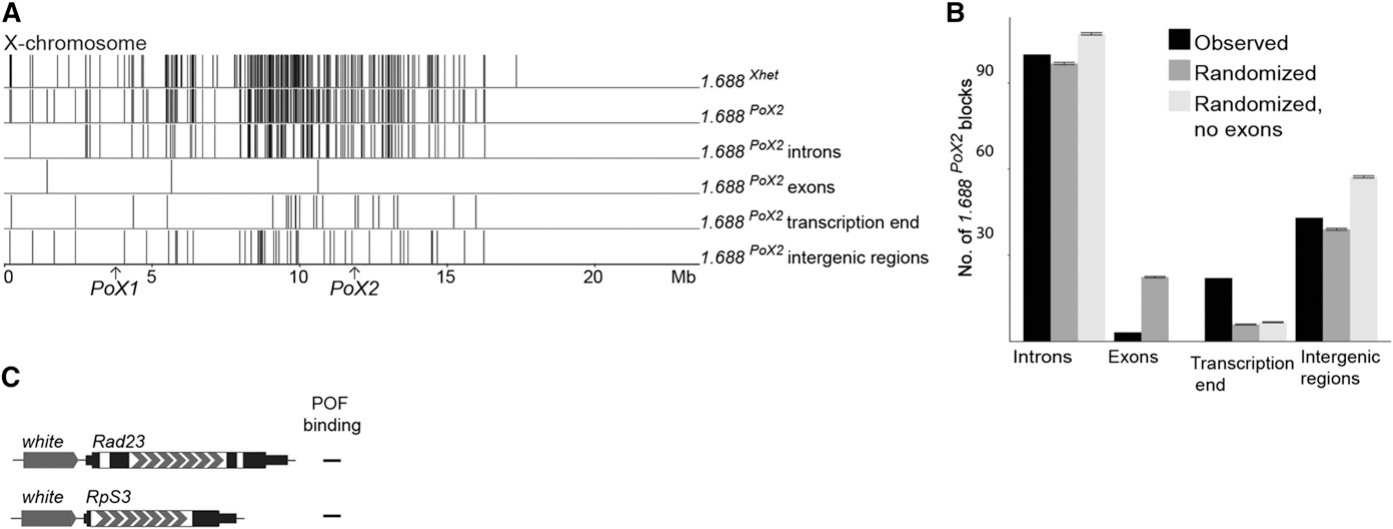
Spatial distribution and locations of the 1.688 satellite. (A) Distribution of 1.688 blocks on the *D. melanogaster* X-chromosome identified with Basic local alignment search tool (BLAST) searches using *1.688^Xhet^* or *1.688^PoX2^* as query sequences. The two top rows show all identified blocks. Blocks in introns, exons, and intergenic regions are shown; blocks within 1000-bp downstream of an annotated transcription end are labeled “transcription end”. (B) Number of *1.688^PoX2^* blocks with locations differing from those expected for a randomized distribution. The error bars indicate 95% C.I.s. Note that the number of blocks located within 1000-bp downstream of a transcription end site is significantly higher than expected. (C) Schematic representation of the transgenic constructs with the *1.688^PoX2^* repeat inserted in the intron and their targeting properties in males and females.

Because the 1.688 satellite is enriched ∼50-fold on the X-chromosome compared to autosomes and was previously suggested to be an X-chromosome identifier (Waring and Pollack 1987; Kuhn *et al.* 2012; Gallach 2014, 2015; Menon *et al.* 2014; Joshi and Meller 2017), we investigated the distribution and location of 1.688 satellite sequences. In addition to the pericentromeric megabase pair block of *1.688^Xhet^*, the 1.688 satellite is heavily enriched along the X-chromosome, with the highest density in the center of the chromosome arm, close to *PoX2* (Figure 5A and Figure S3A in File S1). However, the majority of the repeat arrays are located within introns, and more than would be expected by random chance are located closely downstream (within 1000 bp) of the transcription end (Figure 5B and Figure S3, B and C in File S1). To determine whether an intronic arrangement of the 1.688 satellite could promote targeting, we constructed transgenes with the *8 × 1.688^PoX2^* sequence located in introns. We inserted an intronic repeat in the *RpS3* and the *Rad23* genes, both of which can recruit POF when the repeat is located downstream. These new transgenes did not support targeting (Figure 5C). Taken together, our results strongly suggest that a downstream location of repeats is required for the targeting function, and that transcription of the 1.688 array (as will occur with intronic locations) is not sufficient for targeting.

## Discussion

Mechanisms for recognizing and specifically regulating the X-chromosome are found in many species, where they restore the unequal balance of X-chromosomes between males and females. It is not wholly clear how one specific chromosome is selectively recognized, but repeated sequences including satellites and transposable elements have been suggested to facilitate identification and specific targeting (Ellison and Bachtrog 2013; Menon *et al.* 2014; Joshi and Meller 2017). Here, we provide evidence that when paired with a gene, the 1.688 satellite, which is heavily enriched on the *D. melanogaster* X-chromosome, constitutes a functional targeting element for the chromosome-specific protein POF. Importantly, *PoX2* is a stronger target than any target on the fourth chromosome. Since this X-chromosome targeting of POF to *PoX2* is conserved, it probably confers a selective advantage, which in turn has led to its optimization. The targeting depends on both the repeat’s copy number and its sequence: repeats of *1.688^Xhet^* or *hoppel* do not lead to targeting. On the fourth chromosome, which is rich in repeats, individual repeats and gene plus repeat constellations are not optimized, but the cooperative effect of many genes with suboptimal affinity leads to a high local concentration of POF and thus a fine-tuning of coordinated binding. The repeats on the fourth chromosome only support targeting when under “heterochromatic pressure” and so are nonoptimal as stand-alone POF targets (Johansson *et al.* 2007a). In contrast, the *PoX2* recruitment module is sufficient for stand-alone targeting and thus represents an optimal target. One hypothesis is that targeting depends not only on the specific sequence of the targeted gene, but the combination of the target and the composition of its neighborhood, and that this affects the optimization of different targets. Since the X-chromosome is nonheterochromatic, an optimal target is required for stable targeting in this case. This conclusion is supported by the nature of the observed targeting. *PoX2* is observed in only a fraction of analyzed nuclei, but it always produces a distinct signal without intermediates, suggesting that it acts as a molecular binary switch.

Deletion of the *1.688^PoX2^* array disrupts all binding to *PoX2*, showing that the 1.688 repeat is essential for targeting. In addition, targeting to *PoX1* is decreased dramatically. This suggests a cooperative effect of these X-chromosome targets. The targeting to *PoX1* is rescued by a *PoX2* locus presented in *trans*. Whether this stabilized targeting is mediated via a *trans*-acting RNA intermediate or a three-dimensional chromosomal configuration remains to be determined.

It was recently suggested that the enrichment of 1.688 satellite sequences on the X-chromosome stabilizes the recruitment of the MSL complex (Menon *et al.* 2014; Gallach 2015; Joshi and Meller 2017). This is suggested to be an indirect effect, since 1.688 repeats are dissimilar in sequence to high-affinity sites and because the repeats are not themselves strong targets of the MSL complex (Joshi and Meller 2017). We show here that a 1.688 array placed downstream of a transcribed gene results in this gene becoming a target for POF.

Despite the presence of hundreds of 1.688 satellite copies, *PoX1* and *PoX2* are the only POF-binding sites on the distal X-chromosome. Various factors, including the sequence and number of monomers in an array, might limit the binding of POF to the X-chromosome and thus prevent overexpression of its genes. Another important factor is that the majority of these arrays are located within introns and so cannot act as recruitment elements. This distribution is similar to that expected based on random positioning, but the number of satellites located downstream of genes is significantly higher than expected. The relative overrepresentation of repeat arrays downstream of genes suggests that the presence of such modules may have, or has previously had, a functional advantage. This is further supported by the fact that the POF targeting module has been conserved through evolution, despite the high mutation rate of satellite sequences and the relatively low conservation of the POF protein. Our finding that POF is in close proximity to MSL3 in *D. ananassae* strongly supports a role as part of a cellular dosage compensation system. Furthermore, our results suggest POF to be the primary targeting protein for genes in the vicinity of 1.688 satellite arrays, and it is possible that this targeting may extend to other more distant loci. To summarize, our results provide direct evidence that a repetitive sequence, the 1.688 satellite, plays a role in chromosome-specific targeting.

## Supplementary Material

Supplemental material is available online at www.genetics.org/lookup/suppl/doi:10.1534/genetics.117.300581/-/DC1.
