# Supplementary material for: The X-linked 1.688 Satellite in Drosophila melanogaster Promotes Specific Targeting by Painting of Fourth
Source: Genetics. 2017 Dec 12;208(2):623–32. doi: 10.1534/genetics.117.300581 (PMC5788526; doi:10.1534/genetics.117.300581)
Supplement: Supplementary file 1 [file 623FileS1.pdf]

Supplementary Information for

The X-linked 1.688 satellite in *D. melanogaster* promotes specific targeting by Painting of fourth

Maria Kim, Samaneh Ekhteraei-Tousi, Jacob Lewerentz and Jan Larsson

Department of Molecular Biology, Umeå University, SE-90187 Umeå, Sweden

This PDF includes:

Supplementary Table S1

Supplementary Table S2

Supplementary Table S3

Supplementary Figure S1

Supplementary Figure S2

Supplementary Figure S3

Supplementary Methods

Transgenic constructs

Genomic DNA fragments of *SelG*, *CG1840*, *Rad23*, *Rps3*, *1.688*^{Pox2} were amplified with Phusion DNA polymerase and cloned either directly in *P[w^{+} attB]* or in *pCR®2.1-TOPO* (Thermo Fisher) for more convenient subsequent cloning. Plasmids were verified by sequencing and at least two restriction mappings. PCR primers are listed in Table S1.

Cloning of 2x1.688$^{\#3}$, 8x1.688$^{\#3}$ and 8x1.688^{het} arrays.

To construct the repetitive arrays, monomer segments flanked by *Bam*HI and *Bgl*II and inserted into the *pEX-A2* plasmid were synthesized by Eurofins Genomics (below). In the first cloning step, the plasmid was used to make both the insert and the vector. Fragments were excised with the corresponding restriction enzymes and inserted back into a monomer plasmid digested with *Bgl*II. Thus the resulting plasmid contained a dimer of 1.688 flanked by *Bam*HI and *Bgl*II, and no restriction site in the middle. To obtain an octamer, the cloning procedure was repeated twice. Finally, a dimer and octamer of *1.688*$^{\#3}$ and an octamer of *1.688*^{het} were cloned into *P[w^{+} Rad23]* digested with *Bam*HII downstream of a *Rad23* segment.

Monomer sequence of 1.688$^{\#3}$

```
ggatccatttttttgcaaattttgatgatggtaccccttacaaaaaatgtgaaaatttggccaaaaat
taattttacaaaatccgttttaaagtgataggggttgttagtattggtcataaggagtataaaatggt
aattctttttgctctgtgaccatttttagacaagttatagccaaaaaagccaacttgaaaattgagtt
ttttgccaaaagtagtttccagatttttttgtgaaaaaaatattcggtattttgatcaaaacattgaaa
ataatgactcaaatatggaatgtcatacctcgttgagtttgtttttttaaatcccaatcgatttgcatt
caagtttgggaattctagaatttttagatct
```

Monomer sequence of 1.688^{het}

```
ggatccacattttgcaaattttgatgaccccctccttacaaaaaatgcgaaaattgatccaaaaatt
aatttccctaaatccttcaaaaagtaatagggatcgttagcactggtaattagctgctcaaaacagat
attcgtacatctatgtgaccatttttagccaagttataacgaaaatttcgtttgtaaatatccacttt
tttgcagagtctgtttttccaaatttcggtcatcaaataatcatttattttgccacaacataaaaaat
aattgtctgaatatggaatgtcatatctcactgagctcgtaataaaatttccaatcaaactgtgttca
aaaatggaaattaaatttttttgagatct
```

Cloning of intronic 1.688^{PoX2} transgenes

The *1.688^{PoX2}* array was inserted into the *Hpa*I site of *P[w^{+} Rps3]* by an in-fusion cloning reaction (TBUSA), thus placing the repeats in the first intron of the *Rps3 gene*. Since there is no suitable restriction site in the introns of *Rad23*, it was re-assembled by an in-fusion reaction between 4 segments including the *P[w^{+} attB]*/*Bam*HI vector, two PCR fragments covering the *Rad23* genomic segment and a PCR fragment of *1.688^{PoX2}*. The overhangs were designed so that the *1.688^{PoX2}* was inserted into the third intron of *Rad23*.

Supplementary Table S1. PCR primers used in cloning

Amplified DNA segment	***Primer sequence***
CG1840	ttactagtgcaaaagctctgttttaatgagttc
	aaggatccgtacgattcattgtaagttcggat
SelG	atactagtcagctgttgattgggttattcg
	atggatccgaactcattaaaacagagcttttgc
Rad23	agaaataccgaactacttaaacgga
	ttctccgactcatcctgaaag
RpS3	tccgaaccatgtctgcctagt
	cgtgataccctggatttaagaca
Drosophila yakuba gDNA	tctttgacaaaccgagcgc
	actttcgctctcagaaccga
1.688^{PoX2}	cagatgcttttgacttatttttgc
	cggcggccgcaatttggcggaagattcaaa
Rad23 segment upstream of intronic *1.688^{PoX2}*	gcggccgttactagtggatcagaaataccgaactacttaaacg
	aaataagtcaaaagcatctgtcaacaatatttcttgaccagga
Rad23 segment downstream of intronic *1.688^{PoX2}*	tttgaatcttccgccaaatttcaaaacttatgttgcacattcgt
	cccggtaccgagctcggatcttctccgactcatcctgaaa

1.688^{PoX2} in *RpS3* intron	aatccatcggcttcggagttcagatgcttttgacttatttttgc
	cgcccatctgctttgaggttaatttggcggaagattcaaa
UAS-Hsp70	ttatatttttctcgagcaagcttgcatgcctgcagg
	atatatttatctcgaggcgacgtgttcactttgcttg
Fragment downstream of UAS (5)	tatatatattactagttagcaacggagataactccc
	ttatatttttctcgaggccatgcgtaatcttcttgac
Fragment downstream of UAS (6)	tatatatattactagtccgaacttacaatgaatcgtac
	ttatatttttctcgaggccatgcgtaatcttcttgac

Supplementary Table S2. gRNA oligonucleotides

Primer	***Sequence***
5' forward	cttcgtacgattcattgtaagtt
5' reverse	aaacaacttacaatgaatcgtac
3' forward	cttcgaagtcaagaagattacgca
3' reverse	aaactgcgtaatcttcttgacttc

Supplementary Table S3. PCR primers used in RT-PCR

Primer	***Sequence***
CG1840 3' fw	ccaactcacctctgtgatgc
Rad23 3' fw	taatgtccgcaattatgccc
RpS3 3' fw	agacttagtccctcaccacc
white 3' fw	tcgtgtttactgtttattgcccc
white Δ-wari 3' fw	acctgttcggagtgattagc
Reverse primer	tgaaaagtgaaaggggtggt

Legends for Supplementary Figures

Supplementary Figure S1 ClustalW alignment of a subset of 1.688 satellite sequences based on a gap opening penalty of 15 and a gap expansion penalty of 6.66. Sequences and annotations are according to KUHN *et al.* (2012). The *PoX2* sequence corresponds to the 1.688 sequence of the third repeat in the *PoX2* locus and the Xhet sequence corresponds to the V00225 sequence (X heterochromatin reference sequence) according to KUHN *et al.* (2012).

Supplementary Figure S2 Negative controls verify the specificity of PLA probes in *D. ananassae* males. Polytene chromosomes were hybridised with rabbit anti-POF (top) or goat anti-MSL3 (bottom) antibodies and then with both rabbit plus and goat minus PLA probes. Note the absence of PLA-signal on the chromosomes.

Supplementary Figure S3 Spatial distribution and locations of the 1.688 satellite. (A) Distribution of 1.688 blocks on the different chromosome arms of *D. melanogaster*. 1.688 blocks were identified by BLAST searches using $\mathit{1.688}^{Xhet}$ or $\mathit{1.688}^{PoX2}$ as query sequences. The two top rows show all identified blocks. On the X-chromosome, blocks in introns, exons, and intergenic regions are shown; blocks within 1000 bp downstream of an annotated transcription end are labeled "transcription end". (B) The number of blocks (frequency) and their distance to an annotated transcription end for all intergenic 1.688 blocks on the X-chromosome. The distances are divided in 500 bp bins. The two leftmost bins (0 – 1000 bp) corresponds to those blocks classified as "transcription end" in A. (C) The diagrams show the number of $\mathit{1.688}^{PoX2}$ (left) and $\mathit{1.688}^{Xhet}$ (right) blocks with different locations compared to a randomized distribution on the X-chromosome. The error bars indicate 95% confidence intervals. Note that significantly more blocks than expected are located within 1000 bp downstream of the transcription end site independently of the chosen query sequence.

Figure_S1

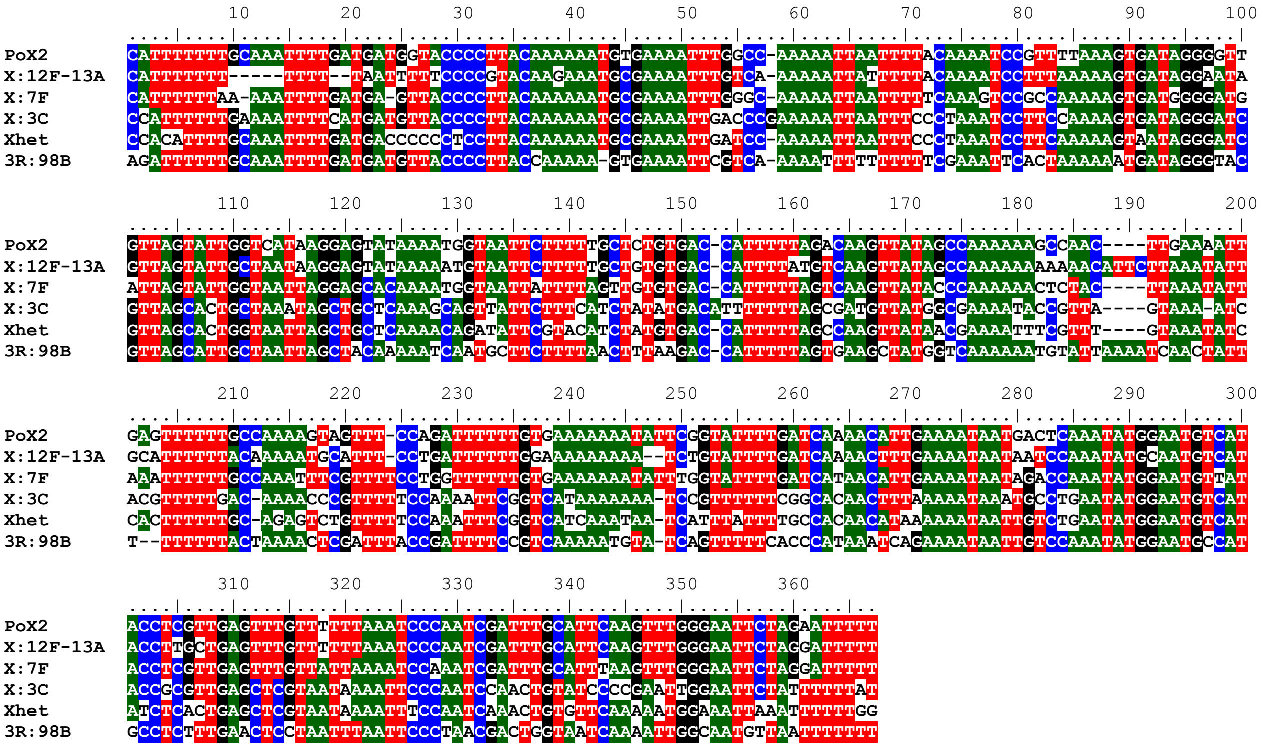
10 20 30 40 50 60 70 80 90 100
PoX2 CATTTTTTTGCAAATTTTGATGATGGTACCCCTTACAAAAAATGTGAAAATTTGGCC-AAAAATTAATTTTACAAAATCCGTTTTAAAGTGATAGGGGTT
X:12F-13A CATTTTTTT-----TTTT--TAATTTTTCCCCGTACAAGAAATGCGAAAATTTGTCA-AAAAATTATTTTTACAAAATCCTTTAAAAAGTGATAGGAATA
X:7F CATTTTTTAA-AAATTTTGATGA-GTTACCCCTTACAAAAAATGCGAAAATTTGGGC-AAAAATTAATTTTTCAAAGTCCGCCAAAAAGTGATGGGGATG
X:3C CCATTTTTTGAAAATTTTCATGATGTTACCCCTTACAAAAAATGCGAAAATTGACCCGAAAAATTAATTTCCCTAAATCCTTCCAAAAGTGATAGGGATC
Xhet CCACATTTTGCAAATTTTGATGACCCCCCTCCTTACAAAAAATGCGAAAATTGATCC-AAAAATTAATTTCCCTAAATCCTTCAAAAAGTAATAGGGATC
3R:98B AGATTTTTTGCAAATTTTGATGATGTTACCCCTTACCAAAAA-GTGAAAATTCGTCA-AAAATTTTTTTTTTCGAAATTCACTAAAAAATGATAGGGTAC
110 120 130 140 150 160 170 180 190 200
PoX2 GTTAGTATTGGTCATAAGGAGTATAAAATGGTAATTCTTTTTGCTCTGTGAC-CATTTTTAGACAAGTTATAGCCAAAAAAGCCAAC----TTGAAAATT
X:12F-13A GTTAGTATTGCTAATAAGGAGTATAAAAATGTAATTCTTTTTGCTGTGTGAC-CATTTTATGTCAAGTTATAGCCAAAAAAAAAAACATTCTTAAATATT
X:7F ATTAGTATTGGTAATTAGGAGCACAAAATGGTAATTATTTTAGTTGTGTGAC-CATTTTTAGTCAAGTTATACCCAAAAAACTCTAC----TTAAATATT
X:3C GTTAGCACTGCTAAATAGCTGCTCAAAGCAGTTATTCTTTCATCTATATGACATTTTTTTAGCGATGTTATGGCGAAAATACCGTTA----GTAAA-ATC
Xhet GTTAGCACTGGTAATTAGCTGCTCAAAACAGATATTCGTACATCTATGTGAC-CATTTTTAGCCAAGTTATAACGAAAATTTCGTTT----GTAAATATC
3R:98B GTTAGCATTGCTAATTAGCTACAAAAATCAATGCTTCTTTTAACTTTAAGAC-CATTTTTAGTGAAGCTATGGTCAAAAAATGTATTAAAATCAACTATT
210 220 230 240 250 260 270 280 290 300
PoX2 GAGTTTTTTGCCAAAAGTAGTTT-CCAGATTTTTTGTGAAAAAAATATTCGGTATTTTGATCAAAACATTGAAAATAATGACTCAAATATGGAATGTCAT
X:12F-13A GCATTTTTTACAAAAATGCATTT-CCTGATTTTTTGGAAAAAAAAA--TCTGTATTTTGATCAAAACTTTGAAAATAATAATCCAAATATGCAATGTCAT
X:7F AAATTTTTTGCCAAATTTCGTTTTCCTGGTTTTTTGTGAAAAAAATATTTGGTATTTTGATCATAACATTGAAAATAATAGACCAAATATGGAATGTTAT
X:3C ACGTTTTTGAC-AAAACCCGTTTTTCCAAAATTCGGTCATAAAAAAA-TCCGTTTTTTCGGCACAACTTTAAAAATAAATGCCTGAATATGGAATGTCAT
Xhet CACTTTTTTGC-AGAGTCTGTTTTTCCAAATTTCGGTCATCAAATAA-TCATTTATTTTGCCACAACATAAAAAATAATTGTCTGAATATGGAATGTCAT
3R:98B T--TTTTTTACTAAAACTCGATTTACCGATTTTCCGTCAAAAATGTA-TCAGTTTTTCACCCATAAATCAGAAAATAATTGTCCAAATATGGAATGCCAT
310 320 330 340 350 360
PoX2 ACCTCGTTGAGTTTGTTTTTTAAATCCCAATCGATTTGCATTCAAGTTTGGGAATTCTAGAATTTTT
X:12F-13A ACCTTGCTGAGTTTGTTTTTTAAATCCCAATCGATTTGCATTCAAGTTTGGGAATTCTAGGATTTTT
X:7F ACCTCGTTGAGTTTGTTATTAAAATCCAAATCGATTTGCATTTAAGTTTGGGAATTCTAGGATTTTT
X:3C ACCGCGTTGAGCTCGTAATAAAATTCCCAATCCAACTGTATCCCCGAATTGGAATTCTATTTTTTAT
Xhet ATCTCACTGAGCTCGTAATAAAATTTCCAATCAAACTGTGTTCAAAAATGGAAATTAAATTTTTTGG
3R:98B GCCTCTTTGAACTCCTAATTTAATTCCCTAACGACTGGTAATCAAAATTGGCAATGTTAATTTTTTT

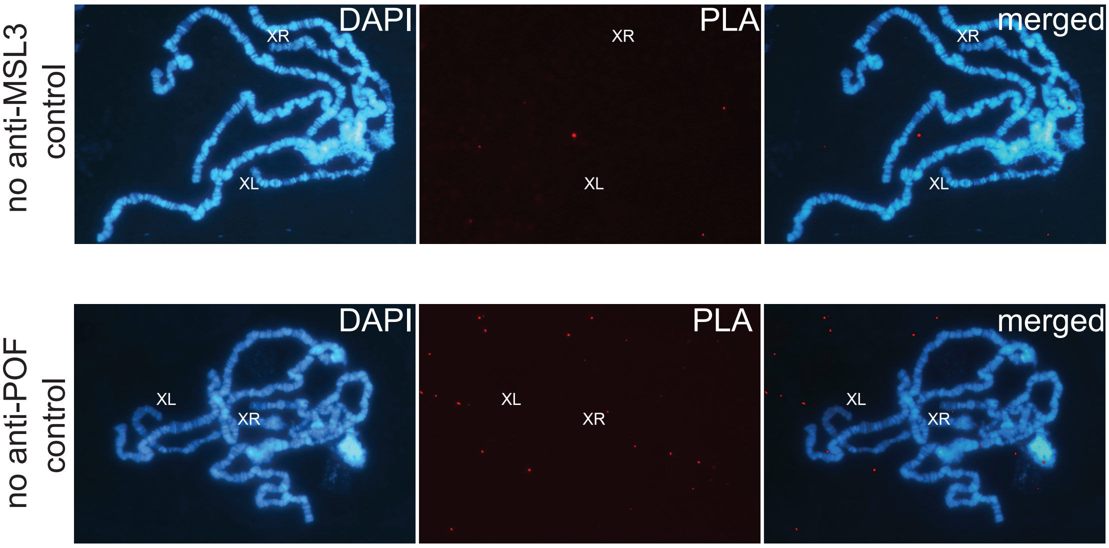
no anti-MSL3
control
DAPI
XR
XL
PLA
XR
XL
merged
XR
XL
no anti-POF
control
DAPI
XL
XR
PLA
XL
XR
merged
XL
XR

Figure_S3

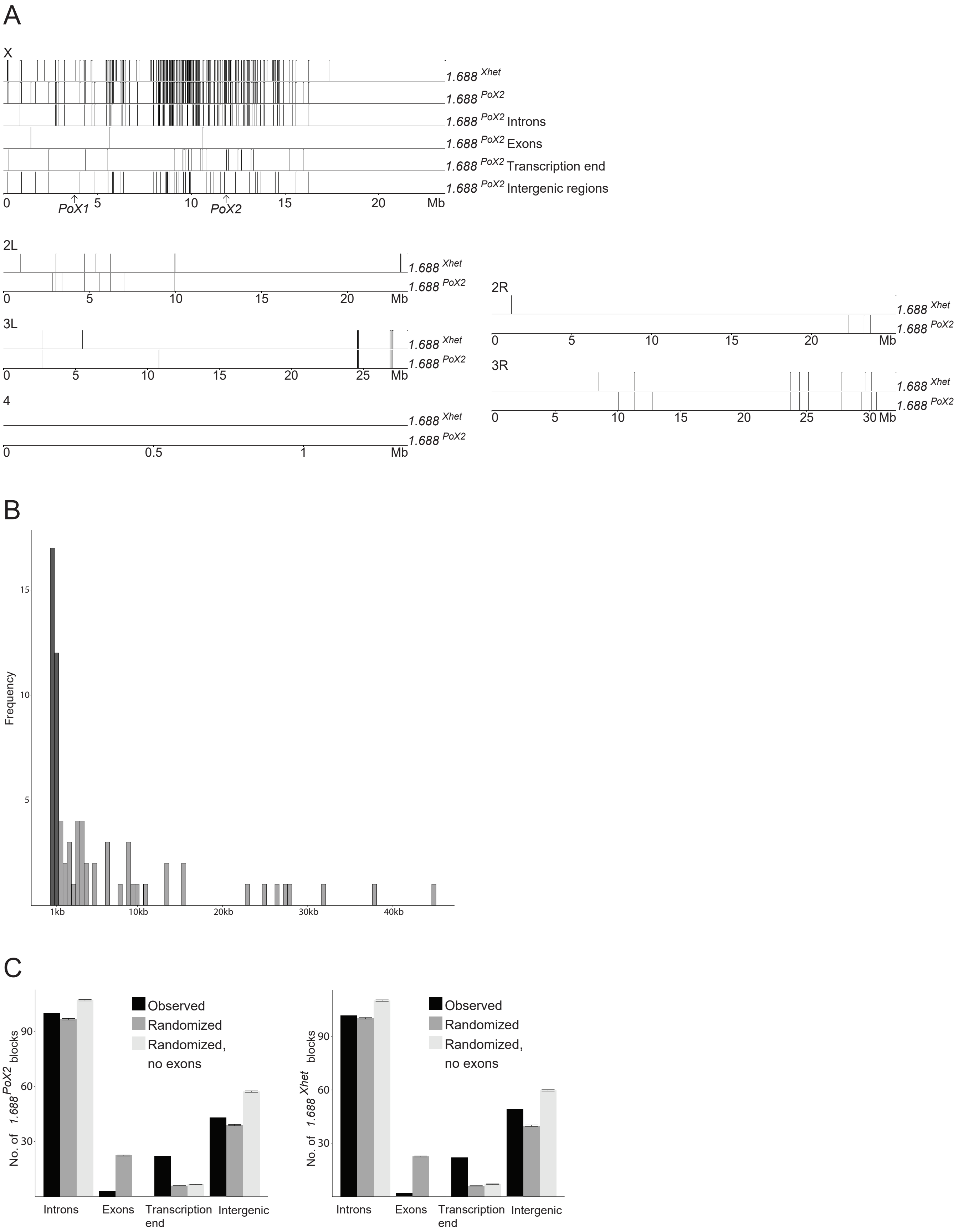
A
X
1.688 Xhet
1.688 PoX2
1.688 PoX2 Introns
1.688 PoX2 Exons
1.688 PoX2 Transcription end
1.688 PoX2 Intergenic regions
0 5 10 15 20 Mb
PoX1
PoX2
2L
3L
4
2R
3R
B
Frequency
1kb 10kb 20kb 30kb 40kb
C
Observed
Randomized
Randomized, no exons
No. of 1.688 PoX2 blocks
No. of 1.688 Xhet blocks
Introns
Exons
Transcription end
Intergenic
